# Rectovaginal Fistula With Double Vagina and Anastomotic Stenosis: A Case Report Following Rectal Cancer Surgery

**DOI:** 10.3389/fonc.2021.549211

**Published:** 2021-02-19

**Authors:** Qiwei Li, Jianhua Sun, Lu Yin, Fu Ji

**Affiliations:** ^1^ Department of General Surgery, Renji Hospital, School of Medicine, Shanghai Jiao Tong University, Shanghai, China; ^2^ Department of Abdominal Surgery, Shanghai Tenth People’s Hospital, School of Medicine, Tongji University, Shanghai, China; ^3^ Department of Gastrointestinal Surgery, Renji Hospital, School of Medicine, Shanghai Jiao Tong University, Shanghai, China

**Keywords:** double vagina, rectovaginal fistula, rectal mucosal advancement flap, transanal endoscopic surgery, anastomotic stenosis

## Abstract

Rectovaginal fistula (RVF) occurs as a result of abnormal epithelialized connections between the rectum and vagina. Rectal cancer surgery remains the major cause of RVF. Here, we report a rare postoperative complication in which a patient with a double uterine and vagina received RVF following rectal cancer surgery. The patient received radiotherapy and developed rectal anastomotic stenosis leading to scar hyperplasia around the fistula, making repair difficult. Complex RVF is prone to release, which despite the multitude of procedures and treatments reported, optimal strategies remain controversial. Our previous studies showed how the use of rectal mucosal advancement flap (RMAF) with transanal endoscopic surgery (TES) can repair mid-low RVF. We successfully repaired RVF and rectal anastomotic stenosis with staging TES in this complex case. This highlights the safety and utility of TES treatment for complex RVF. Further studies are now required to confirm its effectiveness.

## Introduction

Rectovaginal fistula (RVF) is a postoperative complication resulting from the anterior resection of rectal cancer. RVF leads to distressing symptoms, including vaginitis, the passage of flatus/feces through the vagina, and painful skin excoriation. Various procedures and treatments for RVF have been proposed (1–4), with Rectal Mucosal Advancement Flap (RMAF) popular among colorectal surgeons. During this procedure, the patient is placed in the prone Jack-knife position under general anesthetic. Excision and closure of the rectal portion of the fistula are then performed with a vascularized full-thickness rectal flap used to cover the approximated rectovaginal septum on the high pressure side of the fistula (5, 6). The emergence of Transanal Endoscopic Surgery (TES) in the 1980s made minimally invasive surgery for low rectal early tumors and RVF possible (7). Our previous studies highlighted the utility of RMAF with TES as effective treatment strategy for mid-low RVF (8). Complex fistulas are challenging to treat, with the optimal strategy remaining controversial. There are no previous reports of the use of RMAF by TES for the repair of complex RVF. Here, we report a rare complication following rectal cancer surgery, namely an RVF with double vagina. The patient also had rectal anastomotic stenosis making repair more challenging. Treatments were performed with staging TES which successfully cured the patient. We therefore propose that RMAF with TES warrants further investigation as a treatment strategy for complex RVF.

## Case Report

**Figure 1:**
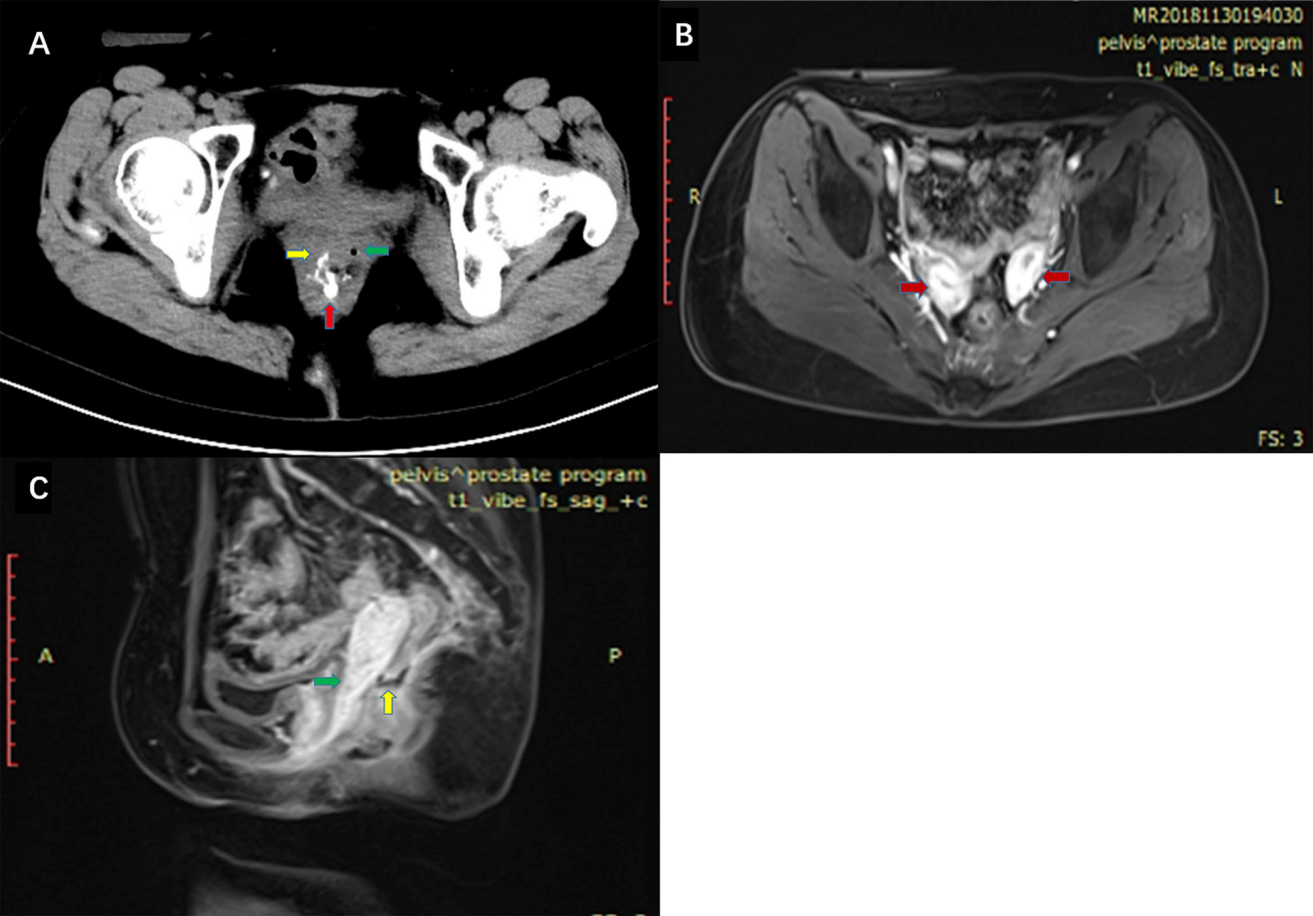
**(A)** High density contrast flow from the rectum into the right vagina in pelvic CT scans after barium enema. Red arrow: rectum. Green arrow: left vagina. Yellow arrow: right vagina. **(B)** Enhanced pelvic MRI. Red arrow: double uterine. **(C)** Enhanced pelvic MRI. Green arrow: vagina. Yellow arrow: fistula between the rectum and vagina.

**Figure 2:**
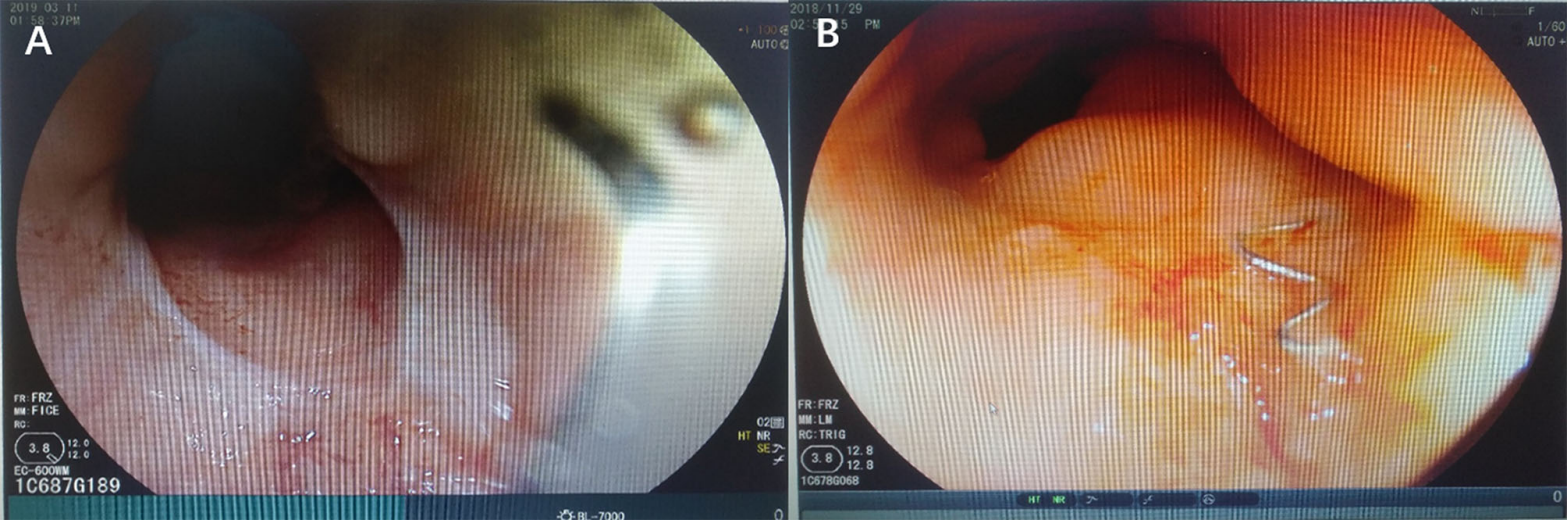
Colonoscopy was performed and identified rectal anastomotic stenosis **(A)** and anastomotic staples **(B)**.

The patient, a female aged 37 years, received neoadjuvant chemoradiotherapy for rectal cancer in May 2017 and underwent laparoscopic anterior resection for rectal cancer and preventive loop ileostomy in July 2017. One week post-surgery, the patient’s vagina displayed fecal water-out. After 1.5 years, she was admitted to our hospital. Abdominal enhanced CT scans indicated that the anterior wall between the rectum and the vagina was not clearly defined. The patient also had a double uterine and vagina. Enhanced pelvic MRI showed the presence of a right mid-low RVF (11–12 o’clock at the lithotomy position) (**Figures 1B, C**). Barium enema showed that the contrast agent entered the vagina through the mid-low rectal fistula (sacrum 4/5 space level) (**Figure 1A**). Colonoscopy showed no obvious fistula, and the rectal anastomosis was narrow (**Figure 2**). Diagnosis: RVF, rectal anastomosis stenosis, and double vagina.

**Figure 3:**
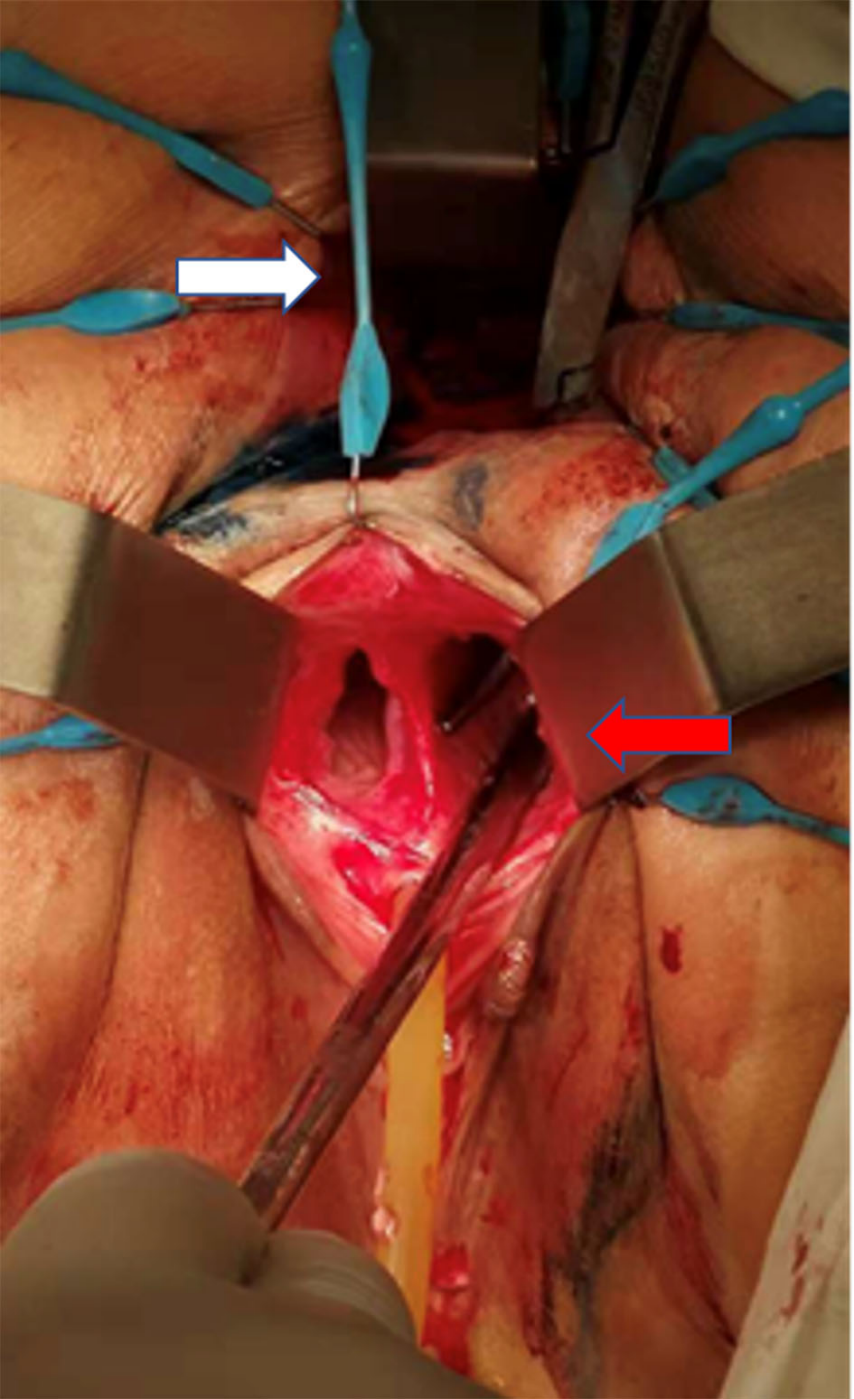
Intraoperative images (the patient was placed in the prone Jack-knife position under general anesthesia.). White arrow: anus. Red arrow: right vagina. The patient had a double vagina. Surgical instruments were placed from the patient’s rectum through RVF into the right vagina.

**Figure 4:**
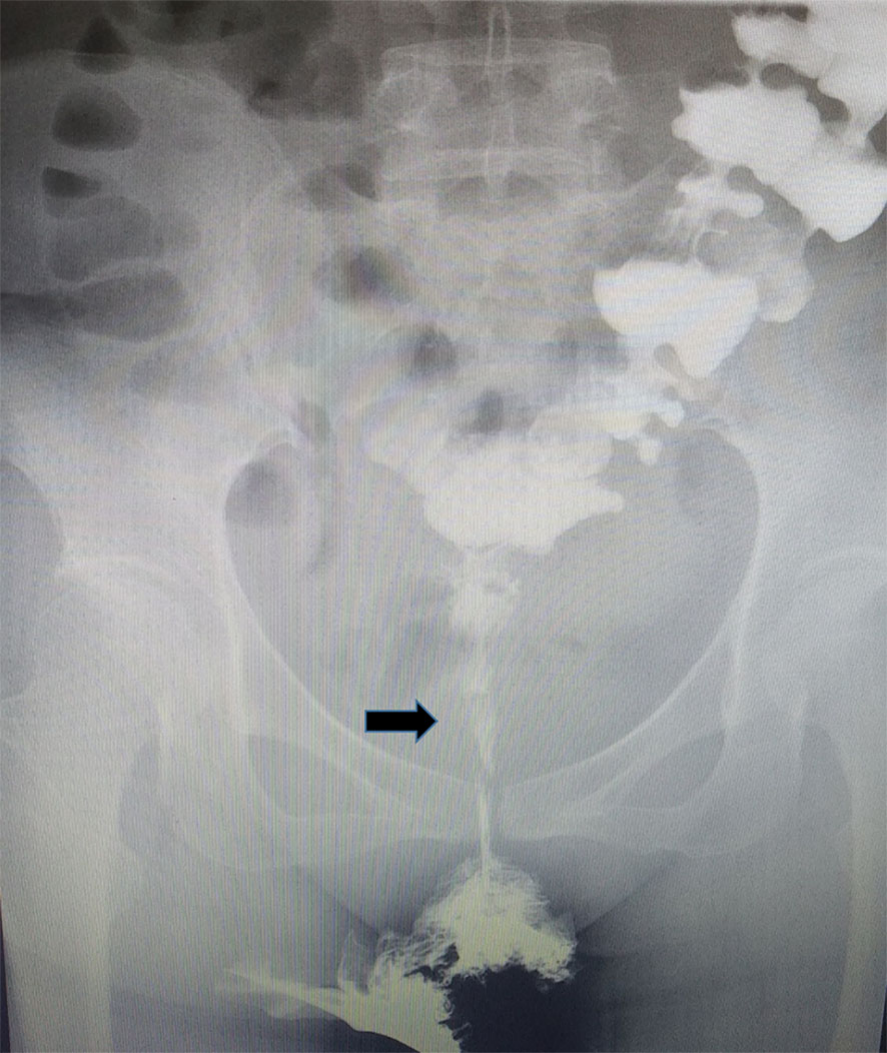
Rectal anastomotic stenosis recurrence was following barium enema. Black arrow: rectal anastomotic stenosis.

The patient received staged minimally invasive surgery which was repaired by RMAF with TES with laparoscopic stoma diversion (single-lumen stoma to prevent the feces flowing to the vagina). Surgery was performed in December 2018. The fistula was present on the right anterior wall of the rectum, approximately 5 cm from the anus, with a diameter of 1.5 cm. The surrounding tissue was tough, and RVF was located in the right vagina (**Figure 3**). After three months, barium enema and pelvic enhancement MRI showed that the RVF healed well. The patient received laparoscopic intestinal stoma closure and rectal anastomosis stenosis incision with TES in March 2019. A single anal canal was placed for support over a two-week period. After 6 months and due to the recurrence of anastomotic stenosis (**Figure 4**), the patient underwent rectal anastomosis stenosis incision, again with TES. Two anal canals were placed for support for ≥10 days. Post-operation, the patient recovered well, with no abdominal pain or fecal water through the vagina. The patient could also eat normally.

## Discussion

RVF is characterized by abnormal epithelialized connections between the rectum and vagina. The condition rarely heals in the absence of interventions (9). The causes of RVF include obstetrical trauma, Crohn’s disease, pelvic irradiation, and post-surgical complications (10). RVF after rectal cancer surgery is more common in the clinic. RVF is divided into simple and complex types. The fistula is considered “complex” when ≥2.5 cm, high, and caused by inflammatory bowel disease or other pelvic processes (diverticulitis), including irradiation (11). Complex RVF repair can easily fail. RVF with a double vagina is extremely rare with no other reports in the literature. Considering the patient’s medical history, RVF caused by the anterior resection of rectal cancer was deemed complex and difficult to repair due to vaginal deformity, chronic tissue inflammation, and scar hyperplasia due to pre-operative radiotherapy and anastomotic stenosis. The optimal treatment strategy to treat this complex disease is currently unclear.

The management of complex RVF is particularly challenging. Various transanal, transvaginal, and perineal surgical approaches have been suggested; however, no optimal operations are currently available. Individualized treatment plans can be formed according to the complexity, location, and size of the fistula (12). We selected repair by RMAF with TES for complex RVF using a wide-based flap of rectal mucosa and the underlying sphincter muscle, which were mobilized and advanced over the internal opening of the fistula. Adequate perfusion and a lack of tension are key to flap success. Radiation also leads to significant tissue damage, fibrosis, and endarteritis, resulting in vascular compromise. Anastomotic stenosis leads to scar hyperplasia around the fistula. The definitive repair of the fistula often requires the interposition of healthy, non-irradiated, well-vascularized tissue to the rectovaginal septum (13). Double vaginal malformation leads to difficulties during surgical exposure and a small operating space. TES has advantages over direct surgery. Under general anesthesia, the TES lens sleeve can be firmly fixed in the anal canal after adequate anal dilatation. High-definition imaging (×6 magnification) can improve exploration in difficult cases. Good visualization allows surgery to be performed with a high degree of precision, minimizing damage to healthy anal and rectal tissue, thereby reducing the risk of postoperative complications. Owing to the high visualization provided by TES, the removal of scar and hardened tissue of the fistula is easier. Foreign bodies such as anastomotic staples should also be removed during operation. A weakness of this technique is its retrospective nature and single-center study. While TES technology requires further assessment, the successful repair of this rare RVF highlights its promise for the treatment of complex RVF.

Ileostomy remains controversial (14). In our experience of patients with large fistulas, obvious scarring and inflammation are common. For relapse-prone patients, prophylactic intestinal fistulas should be administered. The double-lumen stoma performed in this case led to unsatisfactory effects with an intermittent flow of feces to the vagina. We therefore performed double-lumen stoma to the single-lumen to protect the repair.

The appropriate timing of surgical repair is critical to successful healing. In total, 50% of small RVF secondary to obstetric trauma spontaneously heals, and patients should be monitored for at least 6 months (10). Prior to fistula repair, the surgeon must ensure that infections and local inflammation are absent or have been resolved (15). We preoperatively controlled local inflammation through symptomatic treatments, including antibiotic therapy, vaginal irrigation, and intravaginal metronidazole suppositories.

## Conclusions

RVF with double vagina is a rare complication after rectal cancer surgery. The condition is complex and difficult to repair. For this rare RVF, TES showed a favorable effect. This reminds us that the TES procedure for the treatment of RVF and rectal anastomotic stenosis is both safe and feasible. TES technology therefore warrants further application and exploration for the treatment of complex RVF.

## Ethics Statement

Written informed consent was obtained from the individual(s) for the publication of any potentially identifiable images or data included in this article.

## Author Contributions

LY and FJ conceived and revised the manuscript. QL and JS designed and wrote the manuscript. All authors contributed to the article and approved the submitted version.

## Conflict of Interest

The authors declare that the research was conducted in the absence of any commercial or financial relationships that could be construed as a potential conflict of interest.
